# Systemic Burnout in Healthcare: A Conceptual Multilevel Framework of Workforce Erosion and Institutional Fragility

**DOI:** 10.3390/healthcare14131812

**Published:** 2026-06-23

**Authors:** Elena Donisa, Tamara Solange Roșu, Vasile Eduard Roșu, Elena Mihaela Cărăușu

**Affiliations:** 1 Department of Medical Specialties II, Discipline of Nursing, Faculty of Medicine, Grigore T. Popa University of Medicine and Pharmacy, 700115 Iași, Romania; elena.bejenariu-donisa@d.umfiasi.ro; 2 Department of Mother and Child Medicine, Discipline of Pediatrics, Faculty of Medicine, Grigore T. Popa University of Medicine and Pharmacy, 700115 Iași, Romania; eduard.rosu@umfiasi.ro; 3 Grigore T. Popa University of Medicine and Pharmacy, 700115 Iași, Romania; elena.carausu@umfiasi.ro

**Keywords:** burnout, healthcare systems, workforce crisis, human capital, institutional degradation, organizational resilience, post-pandemic healthcare, non-clinical personnel

## Abstract

**Background/Objectives**: Burnout among healthcare professionals has become a major challenge affecting workforce sustainability, quality of care, and organizational performance. Although traditionally conceptualized as an individual response to chronic occupational stress, increasing evidence suggests that burnout is strongly influenced by broader organizational and systemic factors. This article aims to develop a multilevel conceptual framework that explains burnout as a systemic phenomenon emerging from interactions across healthcare structures, institutions, organizations, and individuals. **Methods**: An integrative conceptual synthesis was conducted using literature from healthcare burnout, occupational stress, organizational resilience, workforce sustainability, and health systems research. Relevant theoretical perspectives, including the Maslach Burnout Framework, Job Demands–Resources Model, Conservation of Resources Theory, and organizational resilience literature, were critically examined and integrated to develop a theory-building framework. **Results**: The proposed framework conceptualizes burnout as a dynamic process of pressure transfer operating across five interconnected levels: societal, political, institutional, organizational, and individual. Three central processes are identified: pressure transfer, normalization of exhaustion, and human capital erosion. The model further introduces the concepts of post-pandemic chronicization, invisible burnout, and human infrastructure to explain how prolonged systemic pressures contribute to the normalization and persistence of burnout within healthcare systems. **Conclusions**: Burnout should be understood not only as an individual psychological outcome but also as an indicator of systemic dysfunction. The proposed framework expands existing burnout models by integrating organizational and institutional determinants and provides a foundation for future empirical validation, workforce monitoring, and system-level interventions aimed at strengthening healthcare resilience and sustainability.

## 1. Introduction

Burnout has emerged as a major occupational and organizational challenge in contemporary healthcare. The classical definition describes burnout as a syndrome involving emotional exhaustion, depersonalization or cynicism, and reduced professional efficacy, arising in relation to chronic work stress. This definition remains essential, but the scale, persistence, and post-pandemic amplification of burnout in healthcare indicate that an exclusively individual interpretation is no longer sufficient. Although burnout may coexist with anxiety, depression, psychological distress, and other mental health outcomes, it remains a distinct occupational phenomenon characterized primarily by emotional exhaustion, depersonalization, and reduced professional efficacy. The present framework focuses specifically on burnout while recognizing its interactions with broader psychological and organizational processes [1,2,3,4].

Healthcare workers do not experience exhaustion in a vacuum. Their daily work is shaped by staffing levels, work design, administrative requirements, leadership culture, financing models, digital infrastructure, professional autonomy, public expectations, and political decision-making [5,6,7,8,9,10,11]. Recent evidence from systematic reviews of the pharmacy workforce further supports this systems perspective, demonstrating that workload pressures, organizational support, leadership quality, professional autonomy, and career development opportunities are important determinants of workforce satisfaction, retention, and burnout-related outcomes. The National Academy of Medicine has explicitly framed clinician burnout as a systems problem requiring a systems approach to professional well-being [4,6,12,13]. This article extends that logic by conceptualizing burnout not only as an outcome within healthcare systems, but also as a mechanism through which stressed institutions maintain short-term functionality while consuming human capital.

The COVID-19 pandemic intensified this problem. Multiple reviews documented high levels of burnout and psychological distress among healthcare workers during the pandemic [14,15,16,17,18]. However, the pandemic did not create burnout from nothing. Rather, it exposed pre-existing vulnerabilities: workforce shortages, insufficient surge capacity, operational overload, digital and administrative burden, fragmented governance, and unstable resource allocation. The pandemic can therefore be interpreted as a global stress test that made visible the structural weaknesses already embedded in many healthcare systems [7,8,9,10,11,19,20].

A critical issue is that healthcare systems often respond to burnout through individual-level interventions: resilience workshops, mindfulness programs, stress-management training, or access to counselling. These interventions may be useful for some professionals, but they can become ethically and analytically limited when they imply that individuals must adapt to structurally unsustainable environments. Evidence from intervention research suggests that both individual and organizational strategies can reduce burnout, but organizational change is essential when stressors are embedded in work design [21,22,23,24,25,26].

The present framework does not redefine burnout itself as an institutional phenomenon. Rather, it examines how established burnout processes may emerge within broader organizational and structural environments. The concept of systemic burnout is therefore used as an analytical lens to explore how institutional conditions, workforce dynamics, and governance arrangements may contribute to the development, persistence, and consequences of burnout among healthcare personnel [1,2,3,6].

This article proposes a theoretical framework of systemic burnout in healthcare. The framework integrates psychological burnout theory, the job demands–resources model, conservation of resources theory, demand–control and effort–reward imbalance models, organizational resilience theory, institutional theory, and health workforce policy literature. The aim is to explain how burnout becomes chronic, how it spreads across visible and invisible workforce categories, and how it contributes to gradual institutional degradation [1,2,3,4,5,6,7,8,9,12,15,16,27,28,29,30,31,32,33,34,35,36,37,38,39].

The present article adopts a conceptual and theory-building approach. Rather than generating new empirical data, it integrates findings and theoretical insights from burnout research, occupational stress models, organizational resilience, institutional theory, and healthcare workforce policy. The objective is to develop an integrative framework capable of explaining burnout as a multilevel systemic phenomenon. The framework is intended to generate testable propositions, support organizational analysis, and inform future empirical research on workforce sustainability, institutional resilience, and healthcare system performance [40].

## 2. Conceptual Methodology

This article adopts a theory-building approach based on integrative conceptual synthesis. The purpose of this method is to develop a coherent explanatory framework by bringing together concepts from multiple established studies that have often been examined separately. Rather than generating new empirical data, the article synthesizes existing theoretical and policy perspectives in order to clarify how burnout in healthcare may be understood as a multilevel systemic process [40].

The methodological approach is consistent with theory-building traditions in organizational and health systems research, where conceptual integration is used to identify relationships among constructs, generate explanatory propositions, and guide future empirical investigation. Conceptual synthesis is particularly appropriate when complex phenomena emerge across multiple levels of analysis and cannot be adequately explained by a single theoretical perspective [40].

The conceptual synthesis was informed by a targeted review of literature published in health services research, organizational studies, occupational health, and health policy. Searches were conducted using combinations of terms including burnout, healthcare workforce, workforce shortages, organizational resilience, institutional fragility, health system capacity, and moral distress. Sources were identified primarily through PubMed, Scopus, and Google Scholar.

The conceptual development followed four analytical steps. First, foundational burnout and occupational stress theories were identified, including the Maslach burnout model, the job demands–resources model, conservation of resources theory, the demand-control model, and the effort–reward imbalance model [1,2,3,4,5,6,12,27,28,29]. These theories were selected because they explain how excessive demands, insufficient resources, loss cycles, low autonomy, and inadequate reward contribute to exhaustion, disengagement, and reduced professional efficacy.

Second, the article incorporated literature on moral distress and moral injury in healthcare [17,18,41,42,43,44]. This literature was included because burnout in healthcare is not only a response to workload intensity, but also to repeated situations in which workers are unable to provide care consistent with their professional and ethical commitments. Moral distress is therefore relevant for understanding why burnout may involve loss of meaning, professional identity strain, and withdrawal.

Third, organizational resilience and institutional theory were integrated into the framework [30,31,32,33,34,35,36,37,45]. Resilience theory helps explain how healthcare systems adapt to stress, absorb disruption, and maintain function under variable conditions. Institutional theory helps explain why organizations may adopt formal well-being policies while leaving underlying work conditions unchanged. Together, these perspectives make it possible to distinguish between genuine structural adaptation and symbolic responses to burnout.

Fourth, healthcare workforce policy literature was used to situate burnout within broader system pressures, including workforce shortages, demographic change, professional migration, staff retention, emergency preparedness, and post-pandemic workforce instability [7,8,9,15,16,19,20]. This step was necessary because burnout in healthcare cannot be fully understood without considering the policy and institutional environments that shape staffing, workload, and recovery capacity.

The synthesis was guided by the following conceptual question: how can established theories of burnout, occupational stress, resilience, moral injury, and institutional functioning be integrated to explain the persistence of burnout as a healthcare system problem? From this question, the framework was developed around three dynamic processes: pressure transfer, normalization of exhaustion, and human capital erosion. These processes are conceptual propositions rather than empirically confirmed mechanisms.

**Figure 1:**
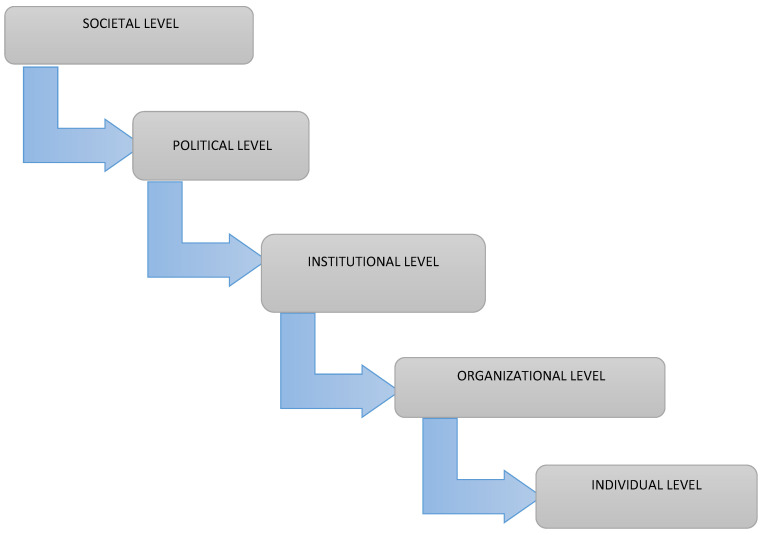
Conceptual representation of how structural pressures may be transferred across societal, political, institutional, organizational, and individual levels and expressed as workforce strain and burnout.

The article uses the term pressure transfer to describe the displacement of unresolved structural and organizational pressures toward healthcare workers. The term human capital erosion refers to the progressive loss of workforce capacity through exhaustion, disengagement, turnover, reduced commitment, and loss of institutional knowledge. The term institutional fragility refers to the reduced ability of healthcare organizations or systems to absorb, adapt to, and recover from operational stress without compromising workforce stability or care delivery. The term post-pandemic chronicization refers to the persistence of exhaustion after the acute crisis phase because of continuing workload, insufficient recovery, and unresolved structural pressures. Finally, invisible burnout refers to burnout affecting non-clinical or less visible personnel whose work sustains healthcare operations but is often underrepresented in burnout research [7,8,32,33] (Figure 1).

**Figure 2:**
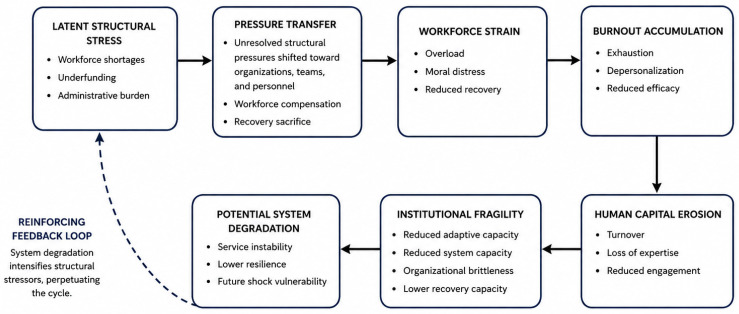
Conceptual pathway linking structural stress, workforce strain, burnout accumulation, human capital erosion, and institutional fragility. The figure represents a theory-building model derived from integrative conceptual synthesis. The relationships shown are proposed conceptual pathways intended for future empirical testing and should not be interpreted as validated causal relationships or quantitative trajectories.

Because this is a conceptual article, the framework should not be interpreted as a tested causal model. The pathways proposed in the article are intended to generate hypotheses, guide future measurement, and support more comprehensive organizational and policy analysis. Empirical studies will be required to examine whether the proposed relationships operate consistently across different healthcare systems, professional groups, and institutional contexts (Figure 2 and Table 1).

## 3. Conceptual Proposition: Burnout as Pressure Transfer

The core proposition of this article is that systemic burnout emerges through pressure transfer. Pressures originating at societal, political, and institutional levels are not fully absorbed by policy, governance, financing, or organizational redesign. Instead, they are progressively displaced downward through organizational hierarchies until they are absorbed by teams and individuals. In this framework, absorption refers to the process through which unresolved organizational or structural demands are managed through increased individual effort, emotional labor, role expansion, or recovery sacrifice rather than through structural adjustment [6,27,28,29,46,47].

At the societal level, healthcare workers face expectations of continuous availability, compassion, performance, and sacrifice. During crises, they may be publicly celebrated as heroes, but symbolic recognition does not necessarily translate into staffing, recovery time, safety, or decision-making authority. Hero narratives may even conceal institutional responsibility by making extraordinary endurance appear normal [10,11,19].

At the political level, healthcare systems are shaped by resource allocation, workforce planning, regulatory decisions, and crisis preparedness. Where policies are reactive, underfunded, or unstable, institutions inherit unresolved pressures. Workforce strategies that do not match demographic change, migration patterns, disease burden, and training capacity create long-term deficits that become operational stressors [7,8,9,11,15,16,20].

At the institutional level, fragmentation, bureaucratic complexity, and chronic underinvestment reduce the capacity of organizations to absorb pressure. Institutional theory helps explain why organizations may adopt formal policies and symbolic programs that display concern for well-being while leaving core work conditions unchanged [37,38,39]. In this way, burnout prevention can become ceremonial rather than transformative.

At the organizational level, pressure is translated into workload, staffing gaps, administrative tasks, shift instability, reduced autonomy, and managerial demands. Leadership may unintentionally amplify pressure by emphasizing productivity, compliance, and throughput without redesigning work processes. Organizational cultures may normalize exhaustion by rewarding availability and framing overwork as professionalism [10,22,23,24,25,26,48,49,50,51,52].

At the team and individual level, pressure finally becomes embodied as fatigue, emotional exhaustion, irritability, detachment, reduced meaning, and intention to leave. The individual worker becomes the final absorptive layer of the system. This does not mean that individual psychology is irrelevant; rather, it means that psychology is the visible surface of a deeper structural process [1,2,3,53].

This pressure-transfer model also explains why burnout can persist despite individual interventions. If the upstream pressure remains unchanged, downstream coping programs may temporarily buffer symptoms without altering the production of exhaustion. A healthcare system may therefore appear to address burnout while continuing to generate it [21,22,24,25,26].

**Figure 3:**
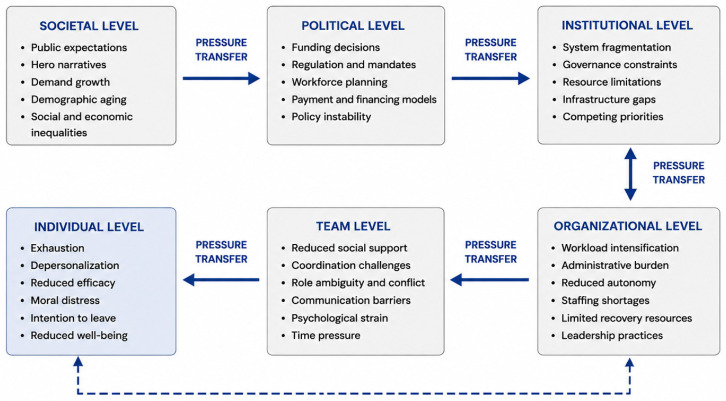
Conceptual representation of the proposed pressure-transfer process across analytical levels of the healthcare system. Structural stress originating at policy and institutional levels is progressively displaced toward organizations, teams, and individuals, where burnout becomes the final absorptive layer of the healthcare system.

Leadership influences may occur at multiple levels, including frontline supervision, middle management, executive leadership, and health system governance. The influence of leadership is also context-dependent and may vary according to organizational resources, regulatory constraints, workforce availability, and broader health system conditions [10,22,25].The proposed pressure-transfer mechanism operating across the different analytical levels of the healthcare system is summarized in Figure 3.

*Pressure Transfer Across Healthcare System Levels*

## 4. Structural Mechanisms Producing Systemic Burnout

In the present framework, structural mechanisms refer to persistent organizational, institutional, or policy-level conditions that systematically shape workforce experiences across time and settings.

Several structural mechanisms contribute to systemic burnout. They are analytically distinct but mutually reinforcing. The first is workforce shortage. When staffing is inadequate, workload rises, recovery time declines, informal support weakens, and the probability of errors or moral conflict increases. Burnout then contributes to attrition, which further worsens staffing. This creates a self-reinforcing loop of human capital depletion [7,8,9,10,11,15,16,20,54,55]. In this framework, human capital depletion refers to the cumulative reduction in workforce capacity through exhaustion, disengagement, turnover, reduced productivity, loss of expertise, and erosion of institutional knowledge.

The second mechanism is chronic administrative and digital burden. Administrative burden refers primarily to documentation, reporting, compliance, and bureaucratic requirements, whereas digital workflow burden refers to workload generated by electronic health records, fragmented digital systems, repetitive data entry, and technology-related inefficiencies. Documentation, electronic health records, quality metrics, compliance procedures, and reporting requirements may improve accountability, but they can also displace time from direct care and professional reflection. Time-motion research has shown that physicians spend substantial time on electronic health record and desk work, contributing to dissatisfaction and burnout [49,50]. Administrative load becomes especially harmful when it is experienced as low-value work disconnected from patient benefit.

The third mechanism is institutional fragmentation. Healthcare delivery depends on coordination among clinical units, administrative offices, laboratories, pharmacies, logistics, public health agencies, insurers, and regulators. Fragmentation increases friction: duplicated documentation, unclear accountability, delays, inconsistent protocols, and communication failures. In fragmented systems, frontline staff often compensate for structural incoherence through personal effort [30,34,35,36,37,38,39,45].

The fourth mechanism is moral constraint. Healthcare workers may know what patients need but lack the resources, time, beds, staffing, or authority to provide it. Such situations can produce moral distress or moral injury, particularly when they recur over long periods [17,18,41,42,43,44]. Moral injury is therefore not only a psychological construct; it is an institutional signal that professional ethics and organizational capacity have become misaligned.

The fifth mechanism is the normalization of exhaustion. Normalization occurs through repeated exposure to excessive workload, cultural reinforcement of self-sacrifice, organizational tolerance of chronic understaffing, and the gradual redefinition of unsustainable working conditions as routine practice. In many healthcare cultures, fatigue is treated as inevitable, overtime as dedication, and self-sacrifice as moral virtue. This cultural pattern reduces the likelihood that exhaustion will be treated as an operational failure. When burnout becomes normal, institutional alarm systems become weaker. What should be a warning sign becomes part of everyday work [10,11,19,26,52,56].

The sixth mechanism is symbolic individualization of responsibility. Organizations may describe burnout using the language of resilience, well-being, and coping while avoiding structural causes such as staffing, workflow, governance, and resource allocation. Resilience is valuable when it means institutional capacity to adapt safely; it becomes problematic when it means asking individuals to endure conditions that should be redesigned [6,12,37,38,39].

These mechanisms convert structural deficits into human depletion. The system continues to function because professionals absorb overload, improvise around inefficiencies, and use personal resources to compensate for institutional gaps. In the short term, this adaptation may protect service continuity. In the long term, it degrades the workforce on which the system depends [6,7,8,11,32,33,55].

## 5. Invisible Burnout: Non-Clinical Personnel as Critical Infrastructure

Burnout research in healthcare has focused primarily on physicians, nurses, emergency staff, and intensive care professionals. This focus is understandable because clinical workers face direct emotional exposure, patient responsibility, and high-risk decision-making. However, it also produces a partial view of healthcare systems [14,15,16,21,23,51,57].

Healthcare institutions are socio-technical systems. In this manuscript, healthcare organizations are conceptualized as socio-technical systems in which human actors, organizational structures, technologies, workflows, and institutional arrangements interact to shape workforce experiences and operational outcomes. Clinical care depends on administrative, logistical, technical, cleaning, maintenance, security, transport, procurement, scheduling, information technology, billing, and operational management functions. These roles are not peripheral. They form the human infrastructure that allows clinical work to occur. The term human infrastructure refers to the workforce capacities, expertise, relationships, tacit knowledge, and coordination functions that enable healthcare systems to operate effectively beyond their formal physical and technological infrastructure [30,31,32,33,34,35].

Non-clinical personnel may experience burnout through different pathways. Their work often combines high pressure with lower symbolic recognition, limited autonomy, insecure status, unclear career development, and exposure to conflict from both staff and patients. They may carry responsibility for operational continuity without equivalent authority over decisions that shape their workload. The category of non-clinical personnel encompasses heterogeneous occupational groups including administrative staff, information technology personnel, operational managers, scheduling coordinators, finance professionals, support services personnel, and other organizational roles whose experiences may differ substantially. Administrative personnel may experience strain through documentation overload, patient-facing conflict, and bureaucratic accountability requirements. Information technology personnel frequently manage system failures, cybersecurity incidents, and digital workflow disruptions under conditions of continuous availability. Logistics and procurement staff may experience chronic pressure related to supply shortages, resource constraints, and operational continuity demands. Environmental services and maintenance personnel may face high workload, infection-related concerns, and limited organizational recognition. These examples illustrate that although burnout manifestations may be similar across occupational groups, exposure pathways and organizational vulnerabilities differ substantially [5,7,8,9,10,11,19,52,55,56].

The invisibility of non-clinical burnout matters because operational dysfunction affects patient care indirectly but powerfully. Administrative delays affect patient flow; logistics failures affect supply availability; IT failures affect documentation and medication processes; cleaning and maintenance failures affect infection prevention and safety; and scheduling failures affect staffing stability. These problems may not be labelled as burnout outcomes, but they can reflect erosion of the human infrastructure of healthcare [30,31,32,33,34,35,45,54,58,59].

Institutional theory helps explain this invisibility. The framework draws primarily upon institutional capacity and organizational resilience perspectives rather than formal institutional isomorphism mechanisms. Professional hierarchies assign higher symbolic value to clinical roles, while non-clinical roles are often described as support functions. Yet support does not mean secondary. In complex systems, invisible infrastructure becomes visible mainly when it fails. A systemic burnout framework must therefore include non-clinical personnel in surveillance, intervention, and workforce policy [37,38,39].

Recognizing invisible burnout also has practical implications. The framework suggests that occupational health programs may benefit from extending screening and support initiatives beyond traditionally studied clinical professions. Workforce planning should include administrative and operational roles. Resilience assessments should measure whether the non-clinical infrastructure is stable enough to support safe clinical delivery. Otherwise, healthcare systems may protect the visible workforce while allowing the invisible workforce to degrade [6,7,8,9,10,11,12,19,20].

**Figure 4:**
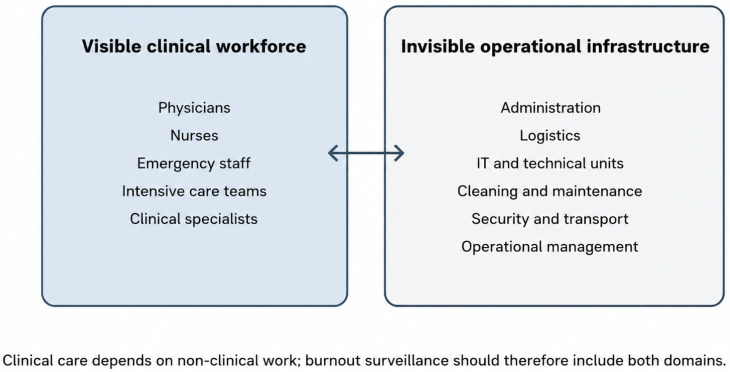
Visible and invisible human infrastructure in healthcare systems. Non-clinical staff constitute operational infrastructure that remains underrepresented in burnout research and workforce protection strategies.

Although non-clinical personnel may experience core dimensions of burnout similar to those observed among clinical staff, their exposure pathways often differ. Administrative overload, coordination responsibilities, technology-mediated workflow demands, organizational mediation roles, and limited visibility within workforce well-being initiatives may create distinctive patterns of occupational strain [5,7,8,9,10,11,19] (Figure 4).

## 6. Post-Pandemic Chronicization and Slow Institutional Degradation

In this framework, chronicization refers to the transition from episodic or acute forms of workforce strain toward persistent, recurrent, and self-reinforcing patterns of occupational exhaustion.

The acute phase of the COVID-19 pandemic produced exceptional stress. Yet the more important theoretical question is why burnout persisted after the acute emergency. If burnout were only a reaction to a temporary crisis, it would be expected to decline once the crisis subsided. Instead, many systems entered a post-pandemic phase characterized by continuing shortages, delayed care backlogs, workforce dissatisfaction, and reduced institutional trust [7,8,9,10,11,14,15,16,19,20].

Post-pandemic burnout differs from acute crisis burnout. Acute burnout is intense, visible, and tied to an identifiable emergency. Chronicized burnout is quieter. It becomes the background condition of work. Workers may no longer describe themselves as being in crisis, but they remain tired, demotivated, detached, and uncertain about the future of their profession [14,15,16,17,18].

Chronicization occurs when there is no structural recovery interval. Structural recovery interval refers to a sustained period during which workload pressures decrease, staffing stabilizes, recovery opportunities are restored, and organizations regain sufficient adaptive capacity to prevent continued workforce depletion. During the pandemic, many workers depleted psychological, physical, and social resources. After the acute phase, delayed care, staffing losses, administrative catch-up, and public frustration continued to generate pressure. The system moved from emergency overload to normalized overload [7,8,9,10,11,19,20].

One consequence is erosion of professional identity. Healthcare work traditionally derives meaning from competence, service, trust, and belonging. When professionals repeatedly work in conditions that prevent them from meeting their own standards, meaning deteriorates. Cynicism and emotional distancing may then become protective strategies rather than moral failures [26,41,42,43,44,53].

A second consequence is symbolic withdrawal. Staff may remain formally employed but reduce emotional investment, initiative, and identification with the institution. This is especially dangerous because it allows organizations to appear staffed while losing engagement, creativity, mentoring capacity, and informal coordination [22,26,48,52].

A third consequence is professional depopulation. Burnout contributes to intention to leave, early retirement, migration, reduction in hours, and career change [9,10,11,20,52,54,55]. Depopulation is not only a numerical problem. It involves loss of institutional memory, team cohesion, tacit knowledge, and intergenerational mentoring.

These processes produce slow institutional degradation. Potential indicators of institutional degradation include increasing turnover, vacancy rates, recruitment delays, declining workforce engagement, reduced organizational learning capacity, operational inefficiencies, and diminished resilience to future disruptions. Unlike sudden collapse, slow degradation is difficult to politicize because services may continue. The institution remains open, patients are still seen, and metrics may be partially maintained. However, the system becomes more brittle, less adaptive, more dependent on extraordinary effort, and less capable of absorbing future shocks [30,31,32,33,34,35,45,54,58,59].

In this sense, systemic burnout is both an outcome and a driver. It is produced by institutional weakness, but once established it accelerates weakness by reducing workforce capacity, increasing turnover, and damaging organizational trust. This feedback dynamic is central to the framework proposed here [6,7,8,26,32,33,52].

**Figure 5:**
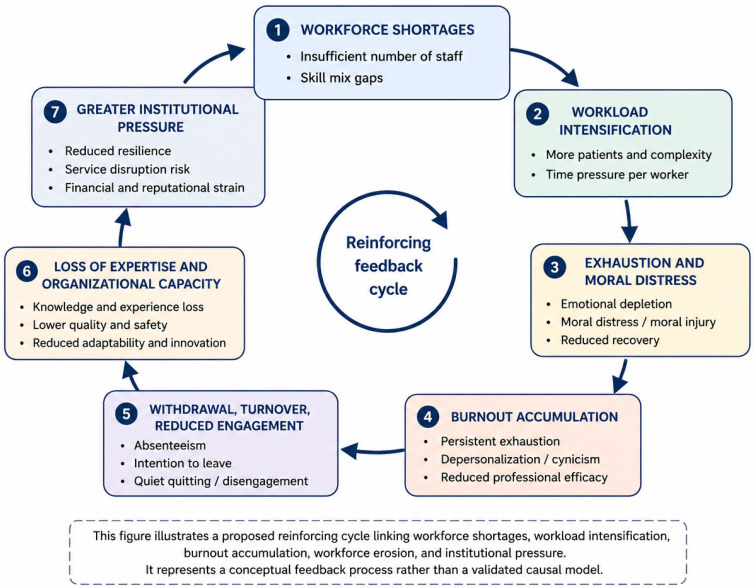
Reinforcing Cycle of Workforce Erosion and Institutional Pressure. Reinforcing cycle linking workforce shortages, burnout accumulation, workforce erosion, and institutional pressure. The figure illustrates a proposed conceptual feedback process rather than a validated causal model.

The reinforcing cycle linking workforce shortages, burnout accumulation, workforce erosion, and institutional pressure is summarized in Figure 5.

## 7. Integrative Multilevel Framework

For conceptual clarity, the framework distinguishes multiple interconnected analytical levels. Although these levels are analytically distinct, they remain interconnected. Political decisions influence institutional capacity, institutional arrangements shape organizational environments, and organizational conditions ultimately affect workforce experiences and burnout outcomes. The political level refers to governmental, regulatory, financing, and policy environments. The institutional level refers to healthcare systems and system-wide organizational arrangements that shape capacity, workforce distribution, and service delivery. Institutional ecology refers to the network of political, regulatory, financial, professional, and organizational structures within which healthcare institutions operate and through which workforce conditions are shaped. The organizational level refers to individual healthcare organizations and their internal structures, leadership practices, workforce management processes, and operational environments [6,30,31,32,33,34,35,36,37,38,39,45].

The integrative framework proposed in this article contains five analytical levels and three dynamic processes. The five levels are societal, political, institutional, organizational, and individual. The three processes are pressure transfer, normalization of exhaustion, and human capital erosion.

At the societal level, healthcare workers are exposed to expectations of compassion, availability, and sacrifice. Public narratives can generate gratitude, but they can also create moral pressure. During crises, heroization may obscure the fact that safe healthcare cannot depend on permanent heroism [10,11,19].

At the political level, decisions regarding funding, staffing pipelines, training capacity, retention strategy, emergency preparedness, and regulation shape the conditions under which institutions operate. Workforce shortages are rarely accidental; they often reflect years of insufficient planning or weak retention policy [7,8,9,10,11,15,16,19,20].

At the institutional level, healthcare systems distribute authority, resources, accountability, and risk. Fragmented institutions may fail to absorb stress, leaving organizations and workers to manage contradictions. Institutional reforms that do not address workforce realities can unintentionally intensify burnout [32,33,34,35,36,37,38,39].

At the organizational level, the framework focuses on work design, leadership, autonomy, administrative burden, team climate, psychological safety, recovery time, and fairness. Organizational strategies are important because they are close enough to daily work to change lived experience, but they require institutional support to be sustainable [10,21,22,23,24,25,26,46,48,49,50].

At the individual level, burnout appears as exhaustion, depersonalization, reduced efficacy, moral distress, identity erosion, and intention to leave. These manifestations are real and clinically important. However, the model treats them as downstream expressions of multilevel dynamics rather than as isolated personal deficits [1,2,3,41,42,43,44,53].

The first dynamic process, pressure transfer, describes the downward displacement of unresolved structural stress. The second, normalization of exhaustion, describes the cultural and institutional process by which overextension becomes routine. The third, human capital erosion, describes the cumulative loss of energy, meaning, competence, trust, institutional memory, and retention capacity [6,7,8,27,28,29].

The framework proposes a potential trajectory: latent structural stress, pressure transfer, operational burnout, chronicization, institutional fragility, and slow degradation. This trajectory is not deterministic. It can be interrupted by structural interventions: staffing redesign, administrative simplification, leadership accountability, workforce retention strategies, psychological safety, investment in non-clinical infrastructure, and participatory governance [7,8,9,10,11,19,20,32,33].

The framework also allows for empirical testing. Future research can examine whether organizations with higher administrative burden, lower autonomy, poorer staffing ratios, and weaker non-clinical infrastructure show higher burnout chronicization and turnover. Longitudinal research can test whether burnout predicts subsequent institutional fragility, not merely individual distress [49,50,51,52,54,58,59].

The framework generates several empirically testable propositions. Future studies may examine whether pressure transfer predicts burnout accumulation, whether burnout predicts human capital erosion, and whether workforce erosion contributes to institutional fragility [40].

### Theoretical Contribution

The present framework extends existing burnout models in five important ways. Unlike existing frameworks that primarily conceptualize burnout as an occupational outcome emerging from work demands and resource imbalances, the present framework proposes a multilevel systems interpretation in which burnout functions simultaneously as a workforce outcome, an institutional signal, and a mechanism through which unresolved structural pressures are transferred and absorbed. The framework therefore extends established burnout models by explicitly linking workforce experiences to processes of institutional fragility, human capital erosion, and post-pandemic chronicization [1,2,3,4,5,6,12,27,28,29,30,31,32,33,34,35,36,37,38,39,45].

First, it conceptualizes burnout as a process of pressure transfer whereby unresolved structural pressures are progressively displaced from societal, political, and institutional levels toward frontline personnel.

Second, it introduces the concept of adaptive sacrifice, distinguishing genuine organizational resilience from system functioning sustained through workforce overextension [30,31,32,33,34,35,45].

Third, it proposes human capital erosion as a cumulative mechanism linking burnout to workforce instability, loss of expertise, and declining organizational capacity [7,8,9,10,11,20,54,55].

Fourth, it introduces institutional fragility as an organizational consequence of persistent burnout and workforce depletion [32,33,34,35].

Finally, the framework explicitly incorporates non-clinical personnel as part of the healthcare workforce infrastructure, extending burnout analysis beyond traditionally studied clinical professions [5,7,8,9,10,11,19].

Proposed Propositions

**P1.**  *Higher structural pressure transfer will be associated with greater workforce strain.*

**P2.**  *Workforce strain will be associated with burnout accumulation.*

**P3.**  *Burnout accumulation will predict human capital erosion.*

**P4.**  *Human capital erosion will contribute to institutional fragility.*

**P5.**  *Institutional fragility will increase vulnerability to future system degradation.*

## 8. Implications for Policy, Management, and Research

The first implication is that burnout prevention should be treated as a governance issue. Ministries, insurers, professional bodies, hospital boards, and regulators influence burnout by shaping staffing, funding, regulation, and accountability. Burnout should therefore be included in health system performance and workforce sustainability metrics [6,7,8,9,10,11,20].

The second implication is that organizations must move from well-being programs to work redesign. Individual support remains necessary, especially for workers experiencing acute distress. However, sustainable prevention requires reducing unnecessary administrative burden, improving staffing adequacy, protecting recovery time, strengthening team support, increasing autonomy, and aligning workload with human capacity [10,21,22,23,24,25,26,46,48,49,50].

The third implication is that healthcare human capital should be treated as critical infrastructure. In this framework, critical infrastructure refers to workforce functions whose disruption would substantially impair healthcare system performance, continuity of operations, coordination, or service delivery. Buildings, beds, equipment, and digital systems are routinely considered infrastructure; the workforce should be treated with the same strategic seriousness. Human capital is not only a cost center. It is the living infrastructure through which care is delivered, coordinated, and improved [7,8,9,10,11,20,32,33,55].

The fourth implication concerns non-clinical personnel. Burnout surveillance should include administrative, technical, logistical, cleaning, maintenance, and operational staff. Excluding these groups produces a distorted understanding of system resilience. If the invisible infrastructure fails, clinical care becomes unstable even when clinical staffing appears adequate [5,7,8,9,10,11,19].

The fifth implication concerns language. Institutions should avoid using resilience language in ways that individualize structural problems. Resilience should mean the capacity of systems to adapt safely and sustainably, not the expectation that individuals will tolerate unsafe or exhausting conditions indefinitely [30,31,32,33,45].

Research should also move beyond cross-sectional prevalence studies. Although prevalence estimates are important, the next phase of research should examine causal pathways, feedback loops, institutional predictors, and longitudinal trajectories. Mixed-methods studies could combine burnout scales, staffing data, turnover data, administrative burden metrics, and qualitative interviews with both clinical and non-clinical staff [40,51,52,54,58,59].

Finally, intervention research should compare individual, organizational, and structural approaches. The key question is not whether mindfulness or counselling can help distressed workers, but whether healthcare systems can redesign work so that fewer workers become chronically distressed in the first place [21,24,25,26,46].

Future intervention research may employ longitudinal, multilevel, comparative, and mixed-method designs to evaluate the relative effectiveness of individual, organizational, and structural interventions across different healthcare settings. Evaluation strategies may also include natural experiments, interrupted time-series analyses, implementation studies, and comparative organizational case studies capable of assessing complex system-level interventions [40].

## 9. Operationalization and Measurement Priorities

Potential dimensions of institutional fragility may include workforce instability, vacancy rates, turnover, recruitment delays, loss of organizational expertise, reduced adaptive capacity, service disruption, operational inflexibility, and diminished resilience during periods of external stress [7,8,9,10,11,20,32,33,54,55].

A systemic model of burnout requires measurement strategies that go beyond individual symptom scales. Instruments such as the Maslach Burnout Inventory remain important for identifying exhaustion, depersonalization, and reduced efficacy [1,2,3,6,12,22,23,27,28,29,48,49,50]. However, if burnout is treated as a system signal, measurement must also include upstream determinants such as staffing adequacy, workload intensity, administrative burden, autonomy, recovery time, leadership quality, psychological safety, and perceived fairness.

At the organizational level, institutions should collect repeated measures rather than isolated annual surveys. Burnout is dynamic. A yearly questionnaire may miss rapid deterioration during staffing crises, policy changes, digital transitions, or seasonal pressure. Short, repeated pulse surveys linked to staffing, absenteeism, turnover, patient flow, and incident data would allow organizations to identify whether exhaustion is becoming chronic [51,52,54,58,59].

At the institutional level, workforce indicators should be interpreted as early-warning signals. High vacancy rates, rising overtime, increasing sick leave, reduced training capacity, and persistent turnover are not merely human resources metrics; they may indicate systemic pressure transfer. A health system that loses staff faster than it can regenerate them is consuming its own operational base [7,8,9,10,11,20,54,55].

Measurement should also include non-clinical personnel. Administrative workers, IT staff, cleaning personnel, logistics teams, maintenance units, and operational managers should be included in workforce well-being dashboards. Their exclusion creates blind spots in resilience assessment and may lead organizations to underestimate operational risk [5,7,8,9,10,11,19].

The framework therefore suggests a dual measurement strategy: symptom measurement at worker level and structural measurement at system level. Burnout scores should be interpreted alongside indicators of demand, resources, governance, staffing, and recovery. Integration may be achieved through organizational dashboards that combine individual burnout measures with workforce, operational, and governance indicators, allowing burnout trends to be interpreted within their broader systemic context. This approach would prevent organizations from treating burnout as a private health problem while ignoring its structural production [1,2,3,6,7,8,9,10,11,12,32,33,51,52,54,58,59].

## 10. Practical Framework for Institutional Intervention

The first practical step is to identify where pressure is generated and where it is absorbed. Operationally, pressure refers to demands that exceed available workforce, organizational, or institutional resources, thereby requiring compensatory effort to maintain system performance. Many healthcare organizations focus on the location where burnout appears-the worker or team-rather than the level at which pressure originates. A systemic diagnostic process should therefore map the path from policy, funding, regulation, and institutional decisions to daily workload and individual exhaustion [6,7,8,9,10,11,30,31,32,33,34,35,45].

The second step is administrative simplification. Healthcare organizations should review documentation requirements, digital workflows, duplicated reporting, and low-value compliance tasks. The aim is not to eliminate accountability, but to reduce work that consumes professional time without improving patient care. Administrative simplification is a structural burnout intervention because it returns time, attention, and meaning to clinical and operational work [22,48,49,50].

The third step is workforce stabilization. Staffing adequacy should not be considered only a budgetary issue. It is a safety, quality, and sustainability issue. Retention strategies should address workload, career development, autonomy, recognition, flexible scheduling, and psychological safety. Recruitment without retention is insufficient because it replaces people while preserving the conditions that drove them away [7,8,9,10,11,20,54,55].

The fourth step is moral repair. When healthcare workers have repeatedly experienced situations in which they could not provide the care they considered ethically appropriate, institutions should not respond only with stress-management language. Moral repair requires acknowledgement, participatory review of constraints, transparent prioritization, and mechanisms through which staff can influence decisions affecting care quality [41,42,43,44].

The fifth step is leadership accountability. Leaders should be evaluated not only on productivity, financial performance, or throughput, but also on workforce sustainability. Departments that function through persistent overtime, high turnover, fear-based cultures, or unaddressed administrative overload should be treated as structurally at risk, even if short-term output appears acceptable [10,22,24,25,26,52].

The sixth step is protection of recovery. A system that has no recovery capacity becomes brittle. Recovery is not a luxury added after work is complete; it is part of the work system. Protected breaks, predictable scheduling, leave access, staffing buffers, and realistic workload planning should be understood as resilience infrastructure rather than as individual benefits [7,8,9,10,11,20,30,31,32,33,34,35,45,54,55].

## 11. Research Agenda

Future research should test the proposed framework empirically. Because several constructs introduced in this framework are conceptual rather than empirically validated, future work should prioritize construct clarification, scale development, and psychometric validation before extensive hypothesis testing [40]. Longitudinal studies are particularly important because systemic burnout develops through accumulation and feedback. Cross-sectional studies can estimate prevalence, but they cannot adequately capture chronicization, pressure transfer, or slow degradation [7,8,9,10,11,14,15,16,51,52,54,58,59].

Comparative studies across healthcare systems could examine whether different governance structures produce different burnout trajectories. For example, systems with stronger workforce planning, lower administrative burden, higher staffing ratios, and better non-clinical infrastructure may demonstrate lower chronicization even when clinical demand is high [7,8,9,10,11,20,32,33,55].

Mixed-methods designs would be especially useful. Quantitative data can identify associations among staffing, workload, burnout, turnover, and safety outcomes. Qualitative data can explain how workers experience pressure transfer, moral constraint, symbolic recognition, and institutional trust. Combining both approaches would strengthen the explanatory value of burnout research [41,42,43,44,51,52,54,58,59].

Research should also include non-clinical staff as a distinct population rather than merging them into general hospital employee categories. Their stressors, resources, and burnout expressions may differ from clinical personnel. Understanding these differences is essential for designing interventions that protect the whole healthcare infrastructure [5,7,8,9,10,11,19].

Finally, intervention studies should compare the effects of individual support, organizational redesign, and system-level policy change. The key research question is not whether burnout can be reduced temporarily, but which interventions interrupt the feedback loop between workforce shortage, workload intensification, exhaustion, withdrawal, and further shortage [10,21,24,25,26,46].

## 12. Relationship to Existing Burnout and Resilience Models

The framework does not replace established burnout models; rather, it extends them. The Maslach model remains central for describing the subjective and professional manifestations of burnout. The job demands-resources model remains central for explaining how excessive demands and insufficient resources produce exhaustion and disengagement. Conservation of resources theory remains useful for understanding depletion cycles. The contribution of the present framework is to situate these mechanisms within a broader institutional ecology, defined here as the network of political, regulatory, financial, professional, and organizational structures within which healthcare institutions operate and through which workforce conditions are shaped [1,2,3,6,12,27,35,36,37,38,39].

In classical occupational models, the organization is often treated as the immediate context of work. In healthcare, however, organizations are themselves embedded in wider political, regulatory, financial, and societal structures. A hospital cannot fully redesign work if it is operating with persistent workforce shortages, unstable financing, delayed reimbursement, fragmented regulation, or national-level training deficits. Systemic burnout therefore requires an analytical scale that extends beyond the employer [7,8,9,10,11,15,16,19,20].

The framework is also related to resilience theory, but it uses resilience cautiously. In resilient healthcare literature, successful performance depends on the capacity to anticipate, monitor, respond, and learn under variable conditions. Yet resilience can be misunderstood when it is reduced to individual endurance. A nurse, physician, administrator, or technician who keeps working despite exhaustion may be resilient in the short term, but a system that depends on such overextension is not resilient in the structural sense [30,31,32,33,34,35,45].

The proposed model distinguishes adaptive capacity from adaptive sacrifice. Adaptive capacity means that systems have buffers, flexibility, learning mechanisms, and recovery resources. Adaptive sacrifice means that workers compensate for missing buffers by giving more time, energy, emotion, and moral effort. Burnout becomes systemic when adaptive sacrifice becomes the routine substitute for adaptive capacity [30,31,32,33,34,35,45].

This distinction is important for policy because it prevents resilience language from being used as a rhetorical shield. A healthcare system should not be considered resilient merely because its workers continue to function under pressure. It should be considered resilient when it can protect care quality and workforce integrity simultaneously [30,31,32,33,34,35,45].

The framework also contributes to institutional theory by showing how formal well-being programs may become symbolic structures if they are not connected to material change. Organizations may adopt visible well-being initiatives to demonstrate legitimacy while leaving staffing, workload, and workflow unchanged [35,36,37,38,39]. Such decoupling can generate cynicism because workers perceive a gap between institutional language and daily experience.

Finally, the model complements quality-of-care frameworks. Donabedian’s distinction between structure, process, and outcome suggests that poor outcomes should be interpreted in relation to organizational structures and processes [45]. Burnout can be read through the same logic: individual exhaustion is an outcome, but its causes may lie in structural and process failures.

## 13. Ethical and Policy Significance

Systemic burnout has ethical significance because it reveals how institutions distribute suffering. When structural problems are transferred downward, those with less authority often bear the highest cost. Frontline clinicians, junior staff, temporary workers, administrative personnel, cleaners, and logistics teams may experience the consequences of decisions made far above them while having limited ability to influence those decisions [7,8,9,10,11,19,20,55].

This distribution of pressure raises questions of procedural justice. If workers are expected to absorb institutional overload, they should also have meaningful participation in decisions about staffing, workflow, safety, and resource allocation. Burnout prevention is therefore not only a technical management issue; it is also a question of voice, fairness, and institutional accountability [10,37,38,39].

The ethical dimension is especially clear in moral injury. When professionals are repeatedly unable to provide care consistent with their values because of organizational constraints, the resulting distress cannot be solved by asking them to become more resilient. Ethical strain requires institutional acknowledgement and structural correction. Otherwise, the language of coping risks becoming a way of normalizing preventable moral harm [17,18,41,42,43,44].

There is also a patient-centered ethical argument. Burnout affects not only workers but also continuity, communication, attention, and safety. A workforce that is emotionally exhausted and institutionally distrustful cannot be expected to sustain high-quality care indefinitely. Protecting workers is therefore part of protecting patients [24,25,47,58,59,60].

Policy makers should treat burnout as a leading indicator rather than a lagging indicator. By the time large numbers of staff leave, reduce hours, or psychologically withdraw, the system has already lost substantial capacity. Earlier indicators—rising exhaustion, declining autonomy, moral distress, administrative overload, and loss of recovery—should trigger intervention before depopulation occurs [7,8,9,10,11,20,32,33,54,55].

The policy significance is particularly strong in countries facing demographic aging, professional migration, and increasing care demand. In such contexts, replacing lost staff may be slow or impossible. Retention becomes as important as recruitment. A health system that cannot retain its workforce cannot maintain access, quality, or crisis readiness [7,8,9,10,11,15,16,20,55].

## 14. Strengths and Limitations of the Framework

The main strength of this article is its integrative scope. It brings together burnout theory, occupational stress models, organizational resilience, institutional theory, moral injury, and healthcare workforce policy into a single conceptual framework. This allows burnout to be interpreted not only as a clinical or psychological issue, but as a marker of institutional functioning [1,2,3,4,5,6,7,8,9,12,15,16,17,18,27,28,29,30,31,32,33,34,35,36,37,41,42,43,44,45].

A second strength is the explicit inclusion of non-clinical personnel. By conceptualizing healthcare as a socio-technical system, the framework expands burnout analysis beyond the visible clinical workforce and highlights the role of hidden operational infrastructures [5,7,8,9,10,11,19].

A third strength is the focus on post-pandemic chronicization. Much pandemic literature understandably emphasized acute distress. This article instead examines the transition from acute overload to normalized exhaustion and slow institutional degradation [7,8,9,10,11,14,15,16,19,20].

The framework also has limitations. It is conceptual and requires empirical validation. Several additional limitations should be acknowledged. First, the framework is intended as a theory-building model rather than a comprehensive explanatory account of burnout. Although it emphasizes structural and institutional influences, burnout remains a multidimensional phenomenon shaped by interactions among organizational, professional, interpersonal, and individual factors. Second, the relative importance of the proposed mechanisms may vary across healthcare professions, organizational settings, and national healthcare systems. Third, some constructs introduced in the framework, including pressure transfer, institutional fragility, adaptive sacrifice, and post-pandemic chronicization, require further conceptual refinement and empirical validation before their measurement properties and boundary conditions can be fully established. Finally, the framework may be vulnerable to conceptual overreach if systemic explanations are interpreted as replacing rather than complementing established occupational, psychological, and organizational perspectives on burnout [1,2,3,4,5,6,7,8,9,12,15,16,27,28,29,30,31,32,33,34,35,36,37,45].

The proposed pathways should be tested using longitudinal, comparative, and mixed-methods designs. The model may also require adaptation across healthcare systems with different financing structures, workforce cultures, and governance arrangements. Although the framework emphasizes structural and institutional influences on burnout, it does not imply that burnout is exclusively determined by system-level factors. Individual vulnerability, personality characteristics, coping resources, professional identity, specialty-specific demands, and national healthcare contexts may also influence burnout trajectories. Consequently, the framework should be interpreted as a systems-oriented explanatory perspective rather than a comprehensive account of all burnout determinants. Future research should examine how structural and individual factors interact across different professional and healthcare settings [40].

Another limitation is that systemic explanations should not erase individual differences. Personal history, health status, social support, professional identity, and coping capacity influence burnout experience. The framework does not deny individual variation; it argues that individual variation occurs within structural conditions that can either protect or deplete workers.

The framework integrates evidence-based observations reported across burnout, workforce, organizational, and health systems studies. However, several proposed relationships represent conceptual extrapolations intended to generate theoretical propositions rather than empirically validated causal pathways. Future research is required to evaluate the strength, direction, and boundary conditions of these relationships [40].

Finally, the framework is intentionally broad. Its value lies in theory-building and policy orientation, but future work should operationalize its constructs into measurable indicators suitable for empirical research and organizational diagnosis [40].

## 15. Conclusions

Burnout remains an important challenge for healthcare systems worldwide. Existing burnout theories have substantially improved understanding of how chronic occupational stress affects healthcare professionals. However, the persistence of burnout across diverse healthcare systems suggests that broader organizational, institutional, and policy-level influences may also warrant attention [1,2,3,4,6,12,14,15,16,21,57].

The present framework proposes that burnout may be understood as a multilevel phenomenon emerging from interactions among societal expectations, policy environments, institutional structures, organizational conditions, and individual experiences. Within this perspective, burnout may serve as an early warning signal of workforce strain, institutional vulnerability, and declining adaptive capacity within healthcare systems [6,7,8,9,10,11,15,16,30,31,32,33,34,35,36,37,38,39,45].

The framework introduces several interconnected concepts, including pressure transfer, normalization of exhaustion, human capital erosion, invisible burnout, post-pandemic chronicization, and institutional fragility. Together, these concepts provide a theoretical basis for examining how workforce depletion may influence organizational resilience and healthcare system sustainability [7,8,9,10,11,30,31,32,33,34,35,36,37,38,39,45].

Particular attention is given to non-clinical personnel, whose contribution to healthcare operations remains insufficiently represented in much of the burnout literature. Understanding workforce sustainability across both clinical and non-clinical roles may contribute to a more comprehensive assessment of healthcare resilience [5,7,8,9,10,11,19].

The proposed relationships should be interpreted as conceptual propositions requiring empirical evaluation. Future longitudinal, comparative, and mixed-methods studies are needed to determine whether the pathways described in this framework are consistently observed across healthcare settings and professional groups [40].

From this perspective, workforce well-being, organizational resilience, and healthcare system sustainability may be viewed as interconnected rather than separate objectives. Further research examining these relationships may contribute to more comprehensive approaches to burnout prevention and health system improvement [7,8,9,10,11,20,32,33,55].

From this perspective, burnout should be understood not only as an occupational health concern but also as a strategic indicator of healthcare system sustainability. Organizations that continuously rely on workforce sacrifice to compensate for structural deficiencies may preserve short-term functionality while gradually undermining their long-term adaptive capacity [7,8,9,10,11,20,32,33,54,55].

The framework suggests that burnout in healthcare may be more fully understood when individual experiences are interpreted within broader organizational, institutional, and systemic contexts [6,30,31,32,33,34,35,36,37,38,39,45] (Table 2).

## Figures and Tables

**Table 1:** Core Constructs of the Framework.

Construct	Definition	Potential Indicators
Pressure Transfer	Displacement of unresolved structural pressures toward frontline personnel	Workload, overtime, staffing deficits
Human Capital Erosion	Progressive loss of workforce capacity	Turnover, absenteeism, reduced engagement
Institutional Fragility	Reduced ability to absorb stress	Vacancy rates, service disruption
Post-Pandemic Chronicization	Persistence of burnout after acute crisis	Longitudinal exhaustion patterns
Invisible Burnout	Burnout among under-recognized non-clinical personnel	Burnout prevalence in operational staff
Adaptive Sacrifice	Maintenance of system functioning through workforce overextension	Overtime dependence, unpaid effort

**Table 2:** Core components of the systemic burnout framework.

Level	Main Pressures	Burnout Pathway	Potential Intervention
Societal	Heroism narratives; expectations of availability	Symbolic pressure and stigma of vulnerability	Public communication that protects rather than romanticizes sacrifice
Political	Funding, workforce planning, emergency preparedness	Under-resourced institutions transfer pressure downward	Long-term workforce strategy and surge capacity
Institutional	Fragmentation, regulation, infrastructure limits	Contradictions become operational strain	Integrated governance and workforce-sensitive regulation
Organizational	Workload, leadership, administrative burden	Daily work exceeds recovery capacity	Work redesign, staffing adequacy, autonomy, psychological safety
Individual/team	Exhaustion, moral distress, withdrawal	Visible expression of systemic pressure	Clinical support plus structural removal of stressors

Note: The framework treats individual burnout symptoms as downstream expressions of multilevel system pressures rather than as isolated personal deficits.

## Data Availability

No new datasets were generated for this article. The study is based exclusively on previously published literature and publicly available sources.
